# GroEL/ES mediated the *in vivo* recovery of TRAIL inclusion bodies in *Escherichia coli*

**DOI:** 10.1038/s41598-018-34090-7

**Published:** 2018-10-25

**Authors:** Zhanqing Wang, Min Zhang, Xin Lv, Jiying Fan, Jian Zhang, Jing Sun, Yaling Shen

**Affiliations:** 1 State Key Laboratory of Bioreactor Engineering, Shanghai Collaborative Innovation Center for Biomanufacturing Technology, East China University of Science and Technology, Shanghai, People’s Republic of China; 2 Shanghai Gebaide Biotechnical Co., Ltd, Shanghai, People’s Republic of China

## Abstract

Inclusion body (IB) formation generates substantial bio-waste in the pharmaceutical industry and remains a major challenge for heterologous protein expression. Although chaperones can be co-expressed to improve soluble protein yield, their contribution to IB processing *in vivo* has not been thoroughly studied. Here, a GroEL-GroES co-expressing strain and a deficient strain were constructed to study the *in vivo* recovery of recombinant human tumor necrosis factor-related apoptosis-inducing ligand (TRAIL). The interaction between GroEL/ES and TRAIL was simulated by molecular docking and identified by co-immunoprecipitation. The *in vitro* cytotoxicity of TRAIL IBs before and after *in vivo* recovery was subsequently determined by MTT assay. Additionally, IB structures were measured by Fourier transform infrared (FT-IR) spectroscopy and fluorescence spectroscopy. The results showed that after *in vivo* refolding, IBs retained lower levels of anti-tumor activity and fewer native-like β-sheet structures. Fewer recoverable polypeptides were trapped in IBs after GroEL/ES co-expression and refolding *in vivo*. Therefore, GroEL/ES mediated the *in vivo* recovery of TRAIL IBs in *Escherichia coli*. These results may identify potential uses for IBs and provide additional insight into the detailed mechanisms of *in vivo* protein recovery.

## Introduction

Inclusion bodies (IBs) are frequently encountered in biochemical and biotechnological research^1,2^. IBs were initially considered bio-waste due to their toxicity to host cells and limited bioactivity, and *in vitro* denaturation and recovery have typically been applied to IBs to obtain active recombinant protein. However, the *in vitro* recovery process is labor intensive and time consuming^3^. In recent decades, IBs were found to possess biological activity and contain correctly folded proteins^4,5^. IBs can act as temporary storage for aggregate-prone polypeptides, and heterologous proteins undergo refolding *in vitro* and *in vivo* following initial incorrect folding in *Escherichia coli*^6^. Therefore, many alternative protocols involving high pressure^7^, alkaline pH conditions^8^ and organic solvents^9^ have been developed to optimize the *in vitro* renaturation process. However, the *in vivo* refolding process for IBs is poorly understood, and investigations into the *in vivo* recovery of IBs are needed.

Tumor necrosis factor-related apoptosis-inducing ligand (TRAIL), also known as human Apo-2 ligand, is a potential protein drug that selectively induces apoptosis in tumor cells without affecting normal cells. Recombinant human TRAIL (rhTRAIL) has been clinically tested^10^ and widely applied in combination therapies. For example, the combination of lovastatin and rhTRAIL has been reported to be a promising strategy for treating glioblastoma^11^. In general, to facilitate medical research, higher soluble TRAIL yield is needed. However, previous studies have shown that the rhTRAIL produced in *E. coli* is mainly in an insoluble form. Molecular chaperones are assumed to be associated with soluble TRAIL production, although this hypothesis has not been confirmed.

Chaperone co-expression is a promising method to improve recombinant protein production, as molecular chaperones have been reported to be involved in protein folding and in the assembly of a variety of substrate proteins^7^. For example, the GroE chaperonins (GroEL-GroES), together constituting a key chaperone system, assist with the expression of functional recombinant proteins in *E. coli*^8^ and *Saccharomyces cerevisiae*^9^ when co-expressed. Moreover, GroE chaperonins help refold denatured peptides into soluble proteins *in vitro*. However, despite improvements in soluble protein production, no universal chaperone system has been established, and the co-expression of molecular chaperones does not always increase protein expression^12,13^. Furthermore, few studies have focused on the role of chaperones in IB processing *in vivo*.

In this study, GroEL/ES co-expressing and deficient strains—GroEL/ES^+^ and GroEL/ES^−^, respectively—were constructed based on the *E. coli* strain C600, and the IB quality and interactions between these chaperones and TRAIL during the *in vivo* recovery process were studied. Specifically, interactions between GroE and TRAIL were simulated by molecular docking and identified by co-immunoprecipitation (co-IP). Additionally, IB activity and their structure in the presence and absence of GroE chaperonins were examined and compared by cytotoxicity assay, Fourier transform infrared (FT-IR) and fluorescence spectroscopy. In summary, the results of this study provide additional insights into the relationship between molecular chaperones and IBs.

## Results

### Role of the GroE chaperonin in rhTRAIL expression and IB refolding

To explore the roles of chaperones in rhTRAIL expression, we utilized the *E. coli* strain C600 (wt) to construct a GroEL/GroES overexpression strain (GroEL/ES^+^) and a GroEL/ES-deficient derivative strain (GroEL/ES^−^). The growth rate of GroEL/ES^+^ was only slightly higher than that of the wt, whereas the growth rate of GroEL/ES^−^ was significantly affected by chaperone deficiency. The growth curves of the strains shown in Supplementary Fig. S1 suggested that loss of the GroE chaperonin resulted in decreased cell viability. The growth of the engineered strains was further investigated by serial dilution spotting (Supplementary Fig. S2). The results show that GroEL/ES was important for both the growth of engineered strains and rhTRAIL expression.

**Figure 1:**
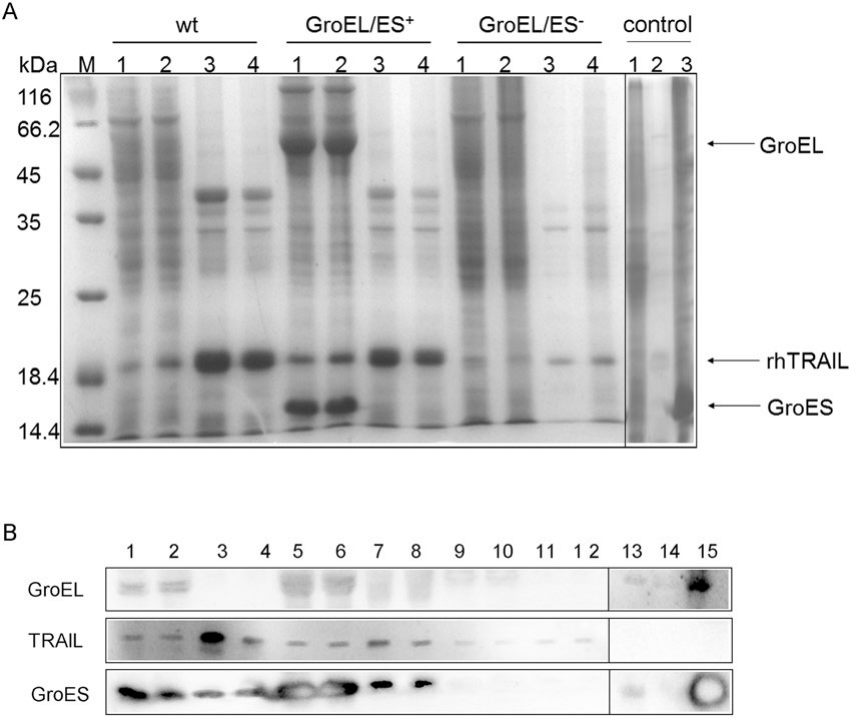
(**A**) SDS-PAGE analysis of rhTRAIL expression in different strains. Lanes are grouped by wt, GroEL/ES^+^ and GroEL/ES^−^. The lanes in each group include (1) the soluble fraction before *in vivo* refolding, (2) the soluble fraction after *in vivo* refolding, (3) the insoluble fraction before *in vivo* refolding, and (4) the insoluble fraction after *in vivo* refolding. The loading control groups were (1) C600, (2) C600-GroE^−^ and (3) C600-GroE^+^. The bands representing GroEL, GroES and rhTRAIL are indicated by arrows. The full-length gel is presented in Supplementary Fig. S4. (**B**) Western blotting to detect rhTRAIL expression in different strains. The lanes in Fig. 1B show the Western blot analysis of corresponding lanes in Fig. 1A.

According to previous studies, rhTRAIL is deposited in both soluble and insoluble forms when expressed in *E. coli*. Here, total protein synthesis was induced in each strain for 4 h and then arrested with chloramphenicol to allow IBs to undergo refolding *in vivo*. Fig. 1B shows the Western blotting results that correspond to Fig. 1A. As shown in Fig. 1, *in vivo* recovery was observed in both the wt and GroEL/ES^+^ strain. However, no significant changes in rhTRAIL levels were observed after refolding in the GroEL/ES^−^ strain. The levels of both soluble and insoluble rhTRAIL were lower in the GroEL/ES^−^ strain than in the wt and GroEL/ES^+^ strains.

To investigate whether GroE chaperonins are involved in the conversion from IBs to sTRAIL, we determined the concentration of rhTRAIL in the soluble and insoluble fractions and calculated the percentage of the soluble form compared to total TRAIL, as shown in Table 1. After *in vivo* recovery for 4 h, soluble TRAIL percentages in the wt and GroEL/ES^+^ strains increased, while TRAIL levels in IBs decreased. The equilibrium between the soluble and insoluble forms shifted toward the soluble form in the presence of GroE. Moreover, the comparison between of the samples obtained before previously and with those obtained after *in vivo* refolding in the wt and GroEL/ES^+^ strains showed that the percentages of soluble TRAIL in the GroEL/ES^+^ strain (23.3% and 34.6%, respectively) were higher than those in the wt strain (16.3% and 22.8%, respectively). There were changes in soluble TRAIL yield between the wt and GroEL/ES^+^ strains (p < 0.05), and the difference was more significant between the wt and GroEL/ES^−^ strains (p < 0.01). These results, as shown in Table 1, indicated that GroE chaperonins improved the yield of soluble TRAIL after protein expression and *in vivo* refolding. The yield of soluble TRAIL in the shake flasks was 78.4 mg/L. The two steps mediated by GroEL/ES may represent an alternative strategy for soluble heterologous protein production compared with that proposed in previous work^14^.

**Table 1:** Concentration and distribution of rhTRAIL between soluble and insoluble forms before and after *in vivo* refolding.

Strain	Before		After	
	Concentration (mg/L)	Percentage (%)	Concentration (mg/L)	Percentage (%)
wt	39.0 ± 5.3	16.3 ± 2.2	54.6 ± 9.1	22.8 ± 3.8
GroEL/ES^+^	52.8 ± 6.3	23.3 ± 2.8	78.4 ± 5.7	34.6 ± 2.5
GroEL/ES^−^	10.9 ± 2.0	12.6 ± 2.3	11.1 ± 2.3	12.8 ± 2.7

IBs produced by the wt, GroEL/ES^+^ and GroEL/ES^−^ strains were designated CT, CGT and DGT, respectively. CT’, CGT’ and DGT’ were the corresponding IBs after *in vivo* refolding. To examine whether the IBs were refolded thoroughly during the *in vivo* process, the *in vitro* recovery potential of IBs after the two-step process was measured^15^. The refolding yield of CT was highest, at 62.5 ± 3.8%, after stepwise dialysis recovery. The refolding yield of CGT was 58.2 ± 3.2%, while that of DGT was only 43.0 ± 4.6%. These findings indicate that IBs can be refolded *in vivo* with chaperonins, but this refolding is incomplete.

### Molecular docking of TRAIL and GroEL

IBs are aggregates of misfolded, partially folded and unfolded intermediates. Based on the results described above, intermediates with native-like structures were contained in TRAIL IBs. As GroEL/GroES chaperones have been reported to promote protein refolding *in vitro* by modulating on-pathway intermediates^16^, chaperonins are assumed to interact with these intermediates in TRAIL IBs. Thus, molecular docking was performed in combination with structural information to predict interactions between GroE chaperonins and native-like polypeptides^17^. The structure of native-like recoverable TRAIL was modeled based on PDB data for native single chains. The spatial structures of GroE and TRAIL were then simulated and docked with the ZDOCK method.

**Figure 2:**
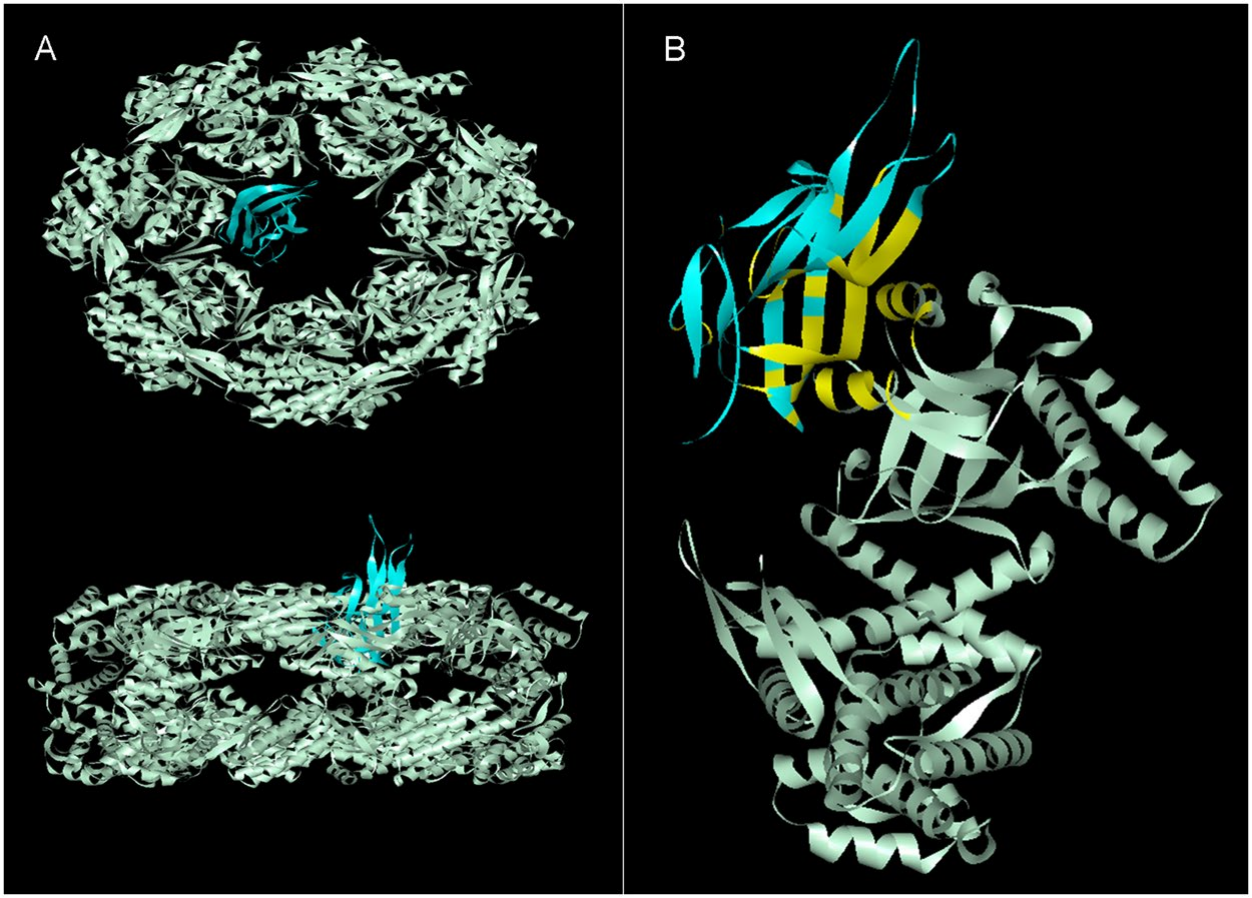
Molecular docking of GroEL (light gray) and TRAIL (blue). (**A**) Top view (top) and side view (bottom) of the docked complex. (**B**) The binding interface (yellow) of the GroEL subunit and single-chain TRAIL.

The simulated complex of the two proteins is shown in Fig. 2. The top view and side view in Fig. 2A indicate that recoverable polypeptides bound to GroEL. To stimulate this interaction in detail, we docked single-chain TRAIL with one of the 14 identical subunits that forms GroEL. As illustrated in Fig. 2B, the binding interfaces on the chaperone involved helix H (234-243 aa) and helix I (257–268 aa) residues in the apical domain of GroEL, which is the binding site of the substrate protein. Based on the docked complex, native-like intermediates were predicted to be capable of binding to GroEL.

### Co-IP analysis of the interaction between GroEL and TRAIL

**Figure 3:**
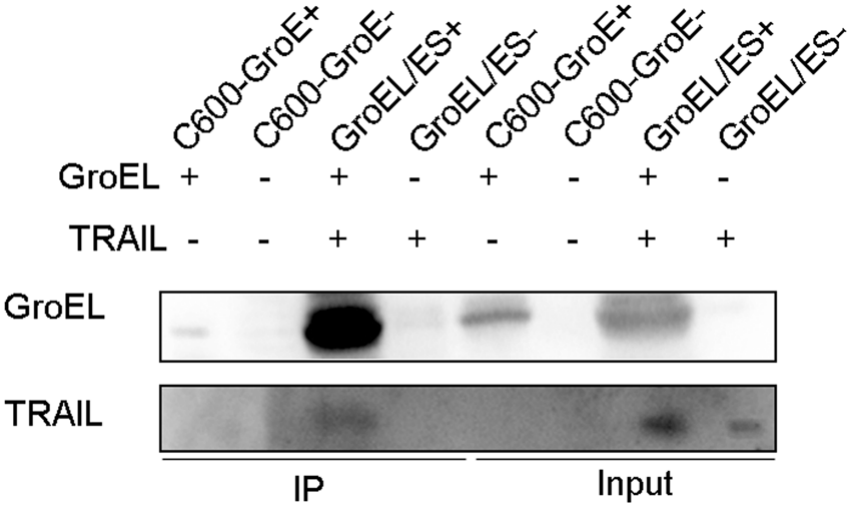
Interaction between GroEL and TRAIL. Strains C600-GroE^+^ and C600-GroE^−^, both constructed in *E. coli* C600, overexpressed GroE and were GroE-deficient, respectively. GroEL/ES^+^ and GroEL/ES^−^ correspond to C600-GroE^+^ and C600-GroE^−^ transformed with the pBV-TRAIL plasmid, respectively. Lysates from different *E. coli* strains were pre-incubated with an anti-GroEL antibody and subsequently bound to protein A/G agarose resin. Samples were eluted with 1× SDS-PAGE loading buffer and subjected to Western blotting with antibodies against GroEL and TRAIL. Samples prepared before and after the co-IP assay are designated input and co-IP, respectively. The cell lysates used for co-IP are presented at the top. The antibodies used for Western blotting are shown on the left.

To determine whether there is a direct physical interaction between GroEL and TRAIL, we conducted a co-IP experiment. Cell lysates were pre-incubated with an anti-GroEL antibody and subsequently mixed with protein A/G agarose resin. Antibody-GroEL-protein complexes were pulled down by the resin, and TRAIL was detected by Western blotting with an anti-TRAIL antibody. As shown in Fig. 3, the antibody-GroEL-TRAIL complex was present in the resulting co-IP solutions from GroEL/ES^+^, revealing the interaction between GroEL and TRAIL in *E. coli*. TRAIL bound to GroEL *in vivo* when the chaperone and TRAIL were co-expressed, but no antibody-GroEL-TRAIL complex was detected when GroEL and TRAIL were expressed separately or were not expressed.

### IB *in vitro* cytotoxic activity before and after *in vivo* refolding

To measure the cytotoxic activity of different TRAIL IBs produced by engineered strains, we studied the inhibition of cell proliferation using the tumor cell line NCI-H460 by using an MTT assay.

**Figure 4:**
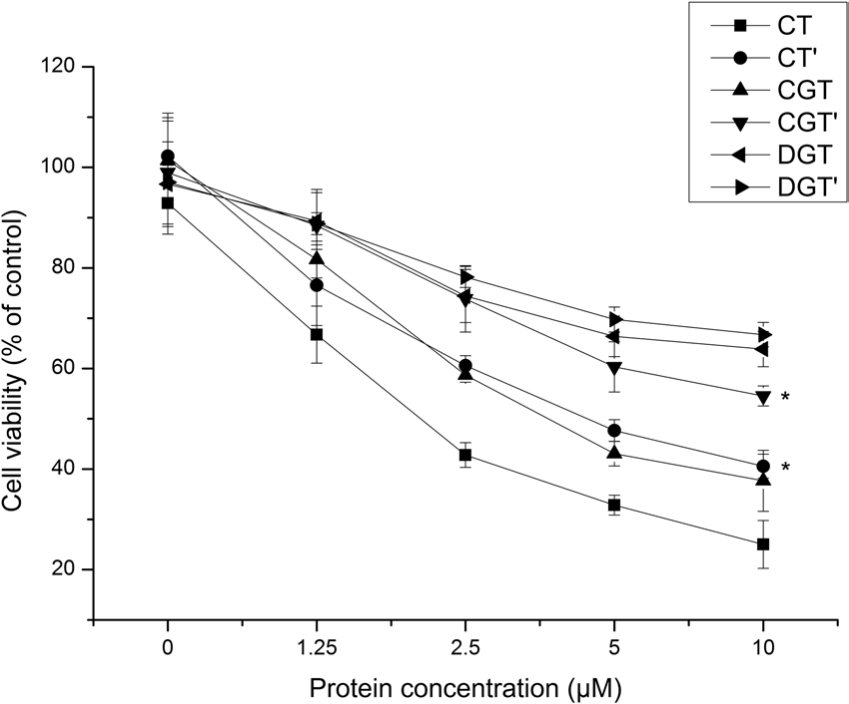
Cytotoxic activity of different TRAIL IBs in the MTT assay. CT, CGT and DGT are IBs produced by the wt, GroEL/ES^+^ and GroEL/ES^−^ strains, respectively. CT’, CGT’ and DGT’ are the corresponding IBs after *in vivo* refolding. The viability of NCI-H460 cells co-cultured with TRAIL IBs at different concentrations (1.25, 2.5, 5 or 10 μM) was measured. Data are presented as the mean ± SD (*n* = 6; *p < 0.05 versus the corresponding group before *in vivo* refolding).

As shown in Fig. 4, all TRAIL IBs suppressed the proliferation of H460 cells, indicating that functional peptides were contained in the IBs^4,5^. Moreover, the inhibition of NCI-H460 cell proliferation by TRAIL IBs was dose-dependent. The cytotoxic activity of IBs was statistically analyzed, and there were no significant changes in cell proliferation were found between DGT and DGT’ (p > 0.05). Therefore, the quantity of active polypeptides in IBs produced by the GroEL/ES^−^ strain was similar before and after *in vivo* refolding. However, the CT’ and CGT’ activities were significantly lower (p < 0.05) than the CT and CGT activities, indicating that the quantity of active polypeptides in IBs produced by the wt and GroEL/ES^+^ strains decreased after GroEL/ES-mediated *in vivo* refolding.

According to the results of the MTT assay, the cytotoxic activity of the IBs produced by the GroEL/ES^−^ strain was much lower than that of the IBs produced by the wt and GroEL/ES^+^ strains, suggesting that the cytotoxic activity of TRAIL IBs is correlated with GroEL/ES expression. In the chaperonin-knockout bacteria, peptides did not fold correctly into their natural or native-like conformations during expression or *in vivo* recovery due to the missing molecular chaperones. Thus, GroE chaperonins are important for the production of functional TRAIL IBs.

Among IBs produced in the presence of GroEL/ES, the anti-tumor activity of CT was the highest, whereas the activity of CGT’ was the lowest and similar to that of DGT and DGT’. This result suggested that higher amounts of functional polypeptides were deposited in the IBs produced by the wt strain before *in vivo* recovery.

### FT-IR analysis of IBs before and after *in vivo* refolding

To determine and analyze the conformation of TRAIL IB particles, solid samples were assessed by FT-IR spectroscopy. The amide I region located between 1700 cm^−1^ and 1600 cm^−1^ in the FT-IR spectra reflects C=O absorption in protein structures. By performing a second derivative treatment on FT-IR spectra in the amide I region, the resulting characteristic absorption peaks can be used to analyze protein secondary structures^18^.

**Figure 5:**
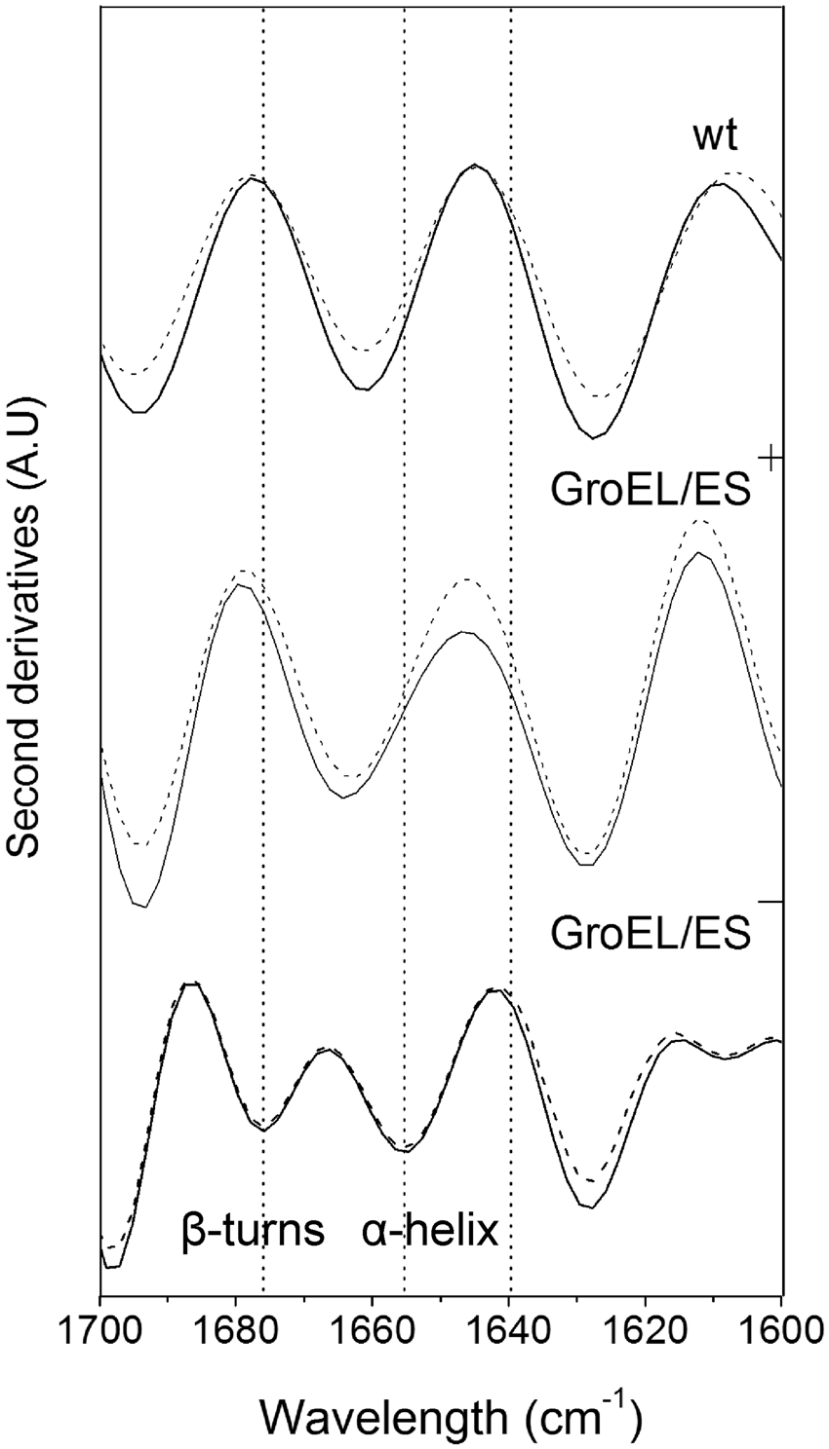
FT-IR analyses of IBs produced by the wt, GroEL/ES^+^ and GroEL/ES^−^ strains. Spectra of samples before (continuous) and after (dashed) *in vivo* refolding are shown. Bands in the wt and GroEL/ES^+^ spectra at 1665 cm^−1^ are attributable to β-turns, whereas the aggregate bands at 1630 cm^−1^ and 1693 cm^−1^ are characteristic of anti-parallel β-sheet intercellular interactions. Bands in the GroEL/ES^−^ spectra at 1654 cm^−1^ and 1678 cm^−1^ were attributable to α-helices and β-turns, and the shoulder at 1640 cm^−1^ indicated intramolecular β-sheet structures.

The second derivative FT-IR spectra collected at 4 cm^−1^ resolution are shown in Fig. 5. As TRAIL is a β-pleated sheet protein^19^, its intramolecular β-sheet structure is attributable to a broad shoulder at approximately 1640 cm^−1^ ^20^. The second-derivative intensity of IBs after *in vivo* recovery observed at 1640 cm^−1^ decreased compared with that of the corresponding IBs before *in vivo* recovery. The results suggested that the native β-sheet component decreased in the samples after four-hour *in vivo* refolding. Contrary to our expectations, the IBs refolded *in vivo* exhibited lower native-like secondary structure levels than did the IBs before refolding. Additionally, differences between the native-like structural contents of CGT and CGT’ were larger than the differences between the other two groups.

Furthermore, the spectra of IBs produced by the wt and GroEL/ES^+^ strains were similar and typical, with major bands at approximately 1630 cm^−1^, 1665 cm^−1^ and 1693 cm^−1^. Bands at 1665 cm^−1^ are attributable to β-turns, whereas the 1630 cm^−1^ and 1693 cm^−1^ bands are characteristic of aggregates caused by intercellular interactions of anti-parallel β-sheets^18^. The second derivative intensity of structural features at 1630 cm^−1^ and 1693 cm^−1^ decreased in each strain after *in vivo* recovery, indicating that the proteins sequestered therein were less aggregated, while a residual intermolecular β-sheet component was maintained. Therefore, chaperonin overexpression facilitates IB recovery *in vivo*.

However, negative minor bands at 1608 cm^−1^, 1654 cm^−1^ and 1678 cm^−1^ were observed in the DGT and DGT’ spectra. Bands at 1654 cm^−1^ and 1678 cm^−1^ in the DGT and DGT’ spectra were attributable to α-helices and β-turns, indicating that more complex, non-native structures were produced in the absence of GroE chaperonins. Moreover, very few differences were observed between the DGT and DGT’ spectra, suggesting that IBs were unaltered during refolding *in vivo* in the absence of GroE chaperonins.

### Fluorescence spectral analysis of denatured IBs *in vitro*

Fluorescence spectral analysis is very useful for detecting changes in protein structure. Tryptophan residues have been widely used as an endogenous probe to study the structures of proteins with intrinsic fluorescence. The bathochromic shift in the maximum fluorescence emission wavelength reflects the degree of conformational change during denaturation. Larger maximum wavelength bathochromic shifts indicate greater amounts of unfolded and denatured protein.

**Figure 6:**
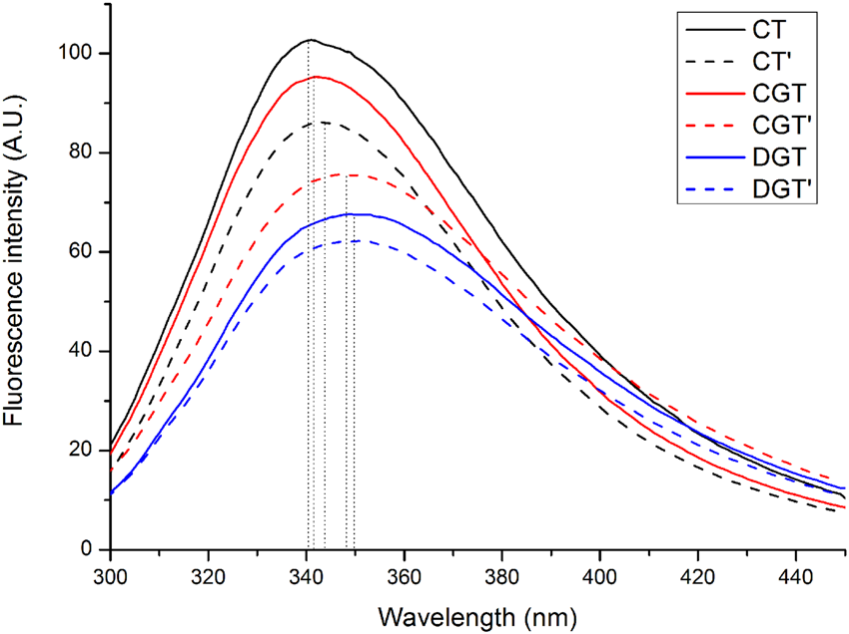
Fluorescence spectra of TRAIL IBs solubilized in denaturing solution. The emission spectra were recorded for IBs produced by wt (black lines), GroEL/ES^+^ (red lines) and GroEL/ES^−^ (blue lines). Spectra of IBs before (continuous) and after (dashed) *in vivo* refolding are shown. The maximum wavelength of each spectrum is shown as a dotted line.

TRAIL IBs were mildly denatured in 2 M urea, which preserves their native-like structure as much as possible for structural characteristic comparisons^21^. The emission peak of tryptophan in native TRAIL is at 330 nm^22^, whereas the emission peak of completely unfolded proteins is at 358 nm^23^. As shown in Fig. 6, the maximum fluorescence emission wavelengths of IBs from the GroEL/ES^+^ and wt strains were approximately 340 nm, suggesting that IBs produced in the presence of chaperonins retained a partial native spatial structure under mild denaturation. However, the IB emission peak increased from 341.8 nm for CGT to 346.8 nm for CGT’. This bathochromic shift and the decreasing fluorescence intensity suggested that the microenvironment of the tryptophan residues changed from the hydrophobic interior to the polar surface. After co-expression and refolding *in vivo* with chaperonins, CGT’ was less compact and denatured more easily than CGT.

However, only slight differences in the maximum wavelengths of DGT and DGT’ were observed, indicating that there are no significant changes in the tryptophan microenvironment. The maximum fluorescence wavelengths of both DGT and DGT’ were approximately 350 nm, i.e., similar to that of completely exposed tryptophan, indicating that the secondary structures of DGT and DGT’ were nearly lost under mild denaturation conditions. Moreover, the fluorescence intensity of DGT and DGT’ produced by the GroEL/ES^−^ strain was lower than the fluorescence intensities of IBs produced by GroEL/ES^+^ and wt strains. Thus, chaperone deletion had a strong effect on the quality of IBs formed during expression.

## Discussion

Previous studies have demonstrated that TRAIL is a promising protein drug for various cancer types^24^. *E. coli* is a common host cell for recombinant TRAIL expression in both biotechnological research and the pharmaceutical industry^25^. Although the soluble expression of several recombinant proteins has been achieved by chaperonin co-expression^26^, IB formation generates substantial bio-waste, which remains a major challenge.

In this study, GroEL/ES co-expression was first confirmed to promote sTRAIL expression in *E. coli*. Then, the *in vivo* recovery of TRAIL IBs and the dynamic transition from insoluble to soluble TRAIL were demonstrated to be related to GroEL/ES co-expression. Thus, the exploitation of GroEL/GroES expression and *in vivo* recovery may represent an alternative method to improve the utilization of bio-resources in TRAIL production.

According to the molecular docking results, recoverable TRAIL bound to GroEL at its apical domain. Peptides with near-native TRAIL structures were then sequestered from the crowded environment inside the cells and separated into a GroEL/ES-constructed hydrophilic chamber^27,28^. GroEL and TRAIL appeared to co-precipitate when they were co-expressed, and their interaction was confirmed by the co-IP assay^29^. These findings indicated that intermediates were likely refolded into soluble proteins by chaperonins.

Because refolding efficiency is closely related to IB structure^8^, the large differences in the *in vitro* refolding yield among IBs were attributable to the co-expression and *in vivo* refolding steps. Therefore, we speculated that fewer recoverable polypeptides were trapped in IBs during expression in the chaperonin co-expression strain, and higher levels of native-like polypeptides in IBs were released during refolding *in vivo*. The results obtained from activity assays and structural analyses (FT-IR and fluorescence spectra) indicated that recoverable TRAIL was first released from IBs by disaggregases such as DnaK and ClpB^30,31^ and then captured by GroEL/ES in the cytoplasm during the *in vivo* renaturation process.

In contrast to the results described by Carriö and Villaverde, the absence of GroEL/ES did not favor the formation of soluble protein in our study^30^. Moreover, both rhTRAIL and sTRAIL levels decreased in a DnaK co-expression strain (data not shown). Thus, chaperones exert different effects on different substrate proteins. This mechanism may be closely associated with the properties of the substrate protein and requires further research. In this study, although GroE chaperonins interacted with TRAIL and played an important role in TRAIL *in vivo* recovery, the observed effects on TRAIL expression/solubility/folding in mutant GroE strains may be a consequence of the misregulation of other proteostasis pathways. However, how multiple chaperone modules cooperate to maintain proteostasis is not yet understood and is the subject of our ongoing research.

In summary, TRAIL IBs formed in *E. coli* were recoverable *in vivo*. GroEL/ES were involved in the expression and refolding of rhTRAIL *in vivo*. Peptides with native-like structures in TRAIL IBs were processed and partially recovered *in vivo* by GroEL/ES. Our results demonstrate that chaperone co-expression combined with *in vivo* recovery is a potential strategy for soluble protein production. Despite the assistance of chaperonins, IBs cannot be completely refolded during *in vivo* recovery. Further study of the *in vivo* recovery process is required to obtain a better yield for the pharmaceutical industry.

## Materials and Methods

### Strains, plasmids and primers

*E. coli* strain C600 was used for strain and plasmid construction in this study. The GroE-deficient strain *E. coli* C600-GroE^−^ was constructed with primer pairs GroE^–^F and GroE^–^R by knocking out the *groel* and *groes* gene using the λ Red recombination system^32^. The genes encoding GroEL and GroES of the *E. coli* strain C600 were cloned with primer pairs GroE^+^-F and GroE^+^-R to create plasmid pGroE. The expression of GroE chaperonins was under the control of their native promoter. The molecular weights of GroEL and GroES were approximately 57 kDa and 10 kDa, respectively. The GroE-overexpression strain *E. coli* C600-GroE^+^ was constructed by transforming pGroE to *E. coli* C600. Then, the wt, GroEL/ES^−^ and GroEL/ES^+^ strains were constructed by harboring plasmid pBV-TRAIL^33^ based on *E. coli* strain C600, C600-GroE^−^ and C600-GroE^+^, respectively. The strains and plasmids used in this study are listed in Table 2. The primers used to construct the strains and plasmids are listed in Table 3.

**Table 2:** List of strains and plasmids used in this study.

Strain or plasmid	Relevant characteristic(s)	Reference or source
**Strain**		
*E. coli* C600	*F-tonA21 thi-1 thr-1 leuB6 lacY1 glnV44 rfbC1 fhuA1 λ-*	Lab collection
C600-GroE^−^	*E. coli* C600 *∆groel/groes*	This study
C600-GroE^+^	*E. coli* C600 harboring pGroE	This study
wt	*E. coli* C600 harboring pBV-TRAIL	Lab collection
GroEL/ES^+^	*E. coli* C600-GroE^+^ harboring pBV-TRAIL	This study
GroEL/ES^−^	*E. coli* C600-GroE^−^ harboring pBV-TRAIL	This study
**Plasmid**		
pBV-TRAIL	Resistance to ampicillin, *pUC ori*	Lab collection
pGroE	Resistance to kanamycin, *pMB1 ori*	This study

**Table 3:** List of primers used in the construction of engineered strains.

Primer	Sequence (5′-3′)
GroE^–^F	CTCCGGCGTCACCCATAACAGATACGGACTTTCTCAAAGGAGAGTTATCAGAGCGATTGTGTAGGCGTG
GroE^–^R	CCAGACATTTCGTCCCGGGGGTTTGTTTATTTCGTCGAGGTGCAGGGCTAACGGCGTACATGGGAATTA
GroE^+^-F	ACGAATTCGTGGTTGATGTCCGATTG
GroE^+^-R	ACTCTAGATTACATCATGCCGCCCATG

### Induction of rhTRAIL and *in vivo* recovery of TRAIL IBs

Bacteria were incubated at 30 °C to an OD_600_ of approximately 0.6, and then the culture temperature was raised to 42 °C to induce rhTRAIL production. After a 4-h induction, half of the cultures were removed before refolding, followed by the addition of chloramphenicol into the remaining cultures at a final concentration of 50 μg mL^−1^. The remaining cultures were further incubated at 30 °C for 4 h to ensure enough time for the *in vivo* recovery of IBs. At least three independent experiments were performed to obtain data for further analysis.

### Separation and purification of soluble TRAIL (sTRAIL) and TRAIL IBs

The harvested cells before and after refolding *in vivo* were sonicated by alternating 5-s cycles of sonication at 200 W and periods of rest. The soluble and insoluble fractions in the cell lysates were separated using Centrifuge 5804 R (Eppendorf, Germany) at 12,000 rpm for 20 min. The insoluble fractions were re-suspended and washed three times in wash buffer (20 mM Tris–HCl, 1 mM EDTA, 1 M NaCl, and 0.4% Triton X-100, pH 8.0)^15^ and then washed another three times with water to obtain purified TRAIL IBs. Soluble TRAIL was purified using a Ni-chelating Sepharose column (GE, USA) as described previously^34^.

### Protein analysis

The obtained samples were analyzed by SDS-PAGE, and the SDS-PAGE gels were scanned and analyzed with a gel analyzer. TRAIL IBs were boiled with 2% SDS for 10 min for solubilization before the concentration was determined. The concentration of all protein samples was determined in triplicate using a BCA protein assay kit (Biomega, USA) with BSA as a standard.

### Molecular docking

The structures of TRAIL and GroEL were modeled based on data from the Protein Data Bank. The entry for TRAIL, GroEL and the subunit of GroEL from *E. coli* was 1DG6, 1OEL and 1KID, respectively^35,36^. The PDB file of single-chain TRAIL was isolated from the 1DG6.pdb using PyMOL software (DeLano Scientific LLC). TRAIL and GroEL were docked using the ZDOCK method.

### Co-immunoprecipitation

Cells were lysed using a one-step bacterial active protein extraction kit (Sangon Biotech, China). The lysates were incubated with anti-GroEL antibody (Abcam, USA) at room temperature for 1 h. Protein A/G Agarose Resin 4FF (Yeasen, China) was extensively washed with washing buffer (0.15 M NaCl, 20 mM Na2HPO4, pH 7.0) and centrifuged at 500 rpm for 1 min. The lysate-antibody mixture was subsequently added to the washed beads, followed by incubation for 30 min at room temperature. The beads were then washed with washing buffer three times and eluted using 25 μL 1× SDS PAGE loading buffer^33^.

### Western blot analysis

Samples were separated using SDS-PAGE and then transferred onto PVDF membranes (Bio-Rad, USA). The membranes were incubated with the primary antibodies (Abcam, USA) at 4 °C overnight and then with the corresponding horseradish peroxidase (HRP)-conjugated secondary antibodies (Abcam, USA) at room temperature for 1 h^34^. Bands were visualized using imaging software (Tanon 4600, Tanon, China).

### *In vitro* cytotoxicity assay

The biological activity of TRAIL IBs was measured by MTT assay. Human non-small cell lung cancer NCI-H460 cells were seeded into 96-well plates at a density of 1 × 10^4^ cells per well. Different concentrations of TRAIL samples were added after cell incubation at 37 °C for 24 h. TRAIL IBs were re-suspended with RPMI-1640 medium (Themofisher, USA), and 100 μL of the suspension was added to each well and incubated with the cells for 48 h^37^. Cells treated with 100 μL of RPMI-1640 medium were used as a control. Then, 20 μL of MTT was added to the wells, and the cells were incubated for another 4 h. Cytotoxicity was determined in triplicate.

### The Fourier transform infrared (FT-IR) spectra

First, 1 mg of dry TRAIL IBs powder and 200 of mg pure KBr were mixed, grinded and tableted. Then, FT-IR spectra were obtained from the tablet using a Spectrum Two spectrometer (PerkinElmer, USA). Each spectrum was obtained between 4000 and 400 cm^−1^ at a resolution of 4 cm^−1^ with 256 scans. The FT-IR spectra was processed with Fourier self-deconvolution using OMNIC software (Themofisher, USA). The second-derivative spectra of TRAIL IBs in the amide I region were obtained using the Savitzky-Golay method^38^.

### Denaturation and recovery of IBs *in vitro*

To maintain more native-like structures when denatured, TRAIL IBs were mildly solubilized in denaturation buffer (20 mM Tris and 2 M urea, pH 8.0) at a ratio of 1:15 (w/v). After *in vitro* denaturation for 8 h, mixtures were centrifuged to obtain the denatured TRAIL samples. The concentration of protein samples was standardized to 1 mg/mL. Denatured samples were dialyzed in sequence in 20 mM Tris (pH 8.0), with 1.5 M/1.0 M/0.5 M/0 M/0 M urea. Samples were adequately dialyzed at each step for 4 h to accomplish the *in vitro* refolding process. The refolded protein samples and the aggregates formed during the refolding *in vitro* process were separated by centrifuging. The refolding yield was calculated using the following formula^39^.

$${\rm{Mass}}\,{\rm{recovery}}\,( \% )={\rm{Mass}}\,{\rm{of}}\,{\rm{refolded}}\,{\rm{TRAIL}}/{\rm{Mass}}\,{\rm{of}}\,{\rm{TRAIL}}\,{\rm{IBs}}\times 100 \% $$

### The fluorescence spectra

The fluorescence emission spectra of denatured TRAIL samples were obtained by using an F-4600 fluorescence spectrophotometer (Hitachi, Japan). Samples were adjusted to 20 μg/mL with the denaturation buffer and excited at 290 nm. Each emission spectra was obtained between 300 nm and 450 nm with a stepsize of 1 nm^21^. Fluorescence spectra were then analyzed with FL Solutions software (Hitachi, Japan).

### Statistical analysis

All data are presented as the mean ± SD (mean ± standard deviation) of at least three independent experiments. Statistical analysis was performed by Student’s *t*-test. Statistical significance was defined as *p < 0.05.

## Electronic supplementary material

Supplementary Information

## Data Availability

All data generated or analyzed during this study are included in this published article.

## References

[1] Overton TW (2014). Recombinant protein production in bacterial hosts. Drug Discov Today..

[2] Roberts CJ (2014). Protein aggregation and its impact on product quality. Curr Opin Biotechnol..

[3] Ami D, Natalello A, Lotti M, Doglia SM (2013). Why and how protein aggregation has to be studied *in vivo*. Microb Cell Fact..

[4] Worrall DM, Goss NH (1989). The formation of biologically active beta-galactosidase inclusion bodies in *Escherichia coli*. Australian J Biotechnol..

[5] Garcia-Fruitos E (2005). Aggregation as bacterial inclusion bodies does not imply inactivation of enzymes and fluorescent proteins. Microb Cell Fact..

[6] Carrio MM, Villaverde A (2001). Protein aggregation as bacterial inclusion bodies is reversible. FEBS Lett..

[7] St John RJ, Carpenter JF, Randolph TW (1999). High pressure fosters protein refolding from aggregates at high concentrations. P Natl Acad Sci USA.

[8] Singh SM, Panda AK (2005). Solubilization and refolding of bacterial IB proteins. J Biosci Bioeng..

[9] Singh SM (2012). Solubilization of inclusion body proteins using n -propanol and its refolding into bioactive form. Protein Expr Purif..

[10] de Miguel D, Lemke J, Anel A, Walczak H, Martinez-Lostao L (2016). Onto better TRAILs for cancer treatment. Cell Death Differ..

[11] Liu PC (2017). Inhibition of NF-kappaB Pathway and Modulation of MAPK Signaling Pathways in Glioblastoma and Implications for Lovastatin and Tumor Necrosis Factor-Related Apoptosis Inducing Ligand (TRAIL) Combination Therapy. PLoS One..

[12] Butz JA, Niebauer RT, Robinson AS (2003). Co-expression of molecular chaperones does not improve the heterologous expression of mammalian G-protein coupled receptor expression in yeast. Biotechnol Bioeng..

[13] Kyratsous CA, Silverstein SJ, DeLong CR, Panagiotidis CA (2009). Chaperone-fusion expression plasmid vectors for improved solubility of recombinant proteins in *Escherichia coli*. Gene..

[14] Zhang M, Wang Z, Chi L, Sun J, Shen Y (2018). Enhanced production of soluble tumor necrosis factor-related apoptosis-inducing ligand in *Escherichia coli* using a novel self-cleavable tag system Fh8-DeltaI-CM. Protein Expr Purif..

[15] Fan J, Wang Z, Huang L, Shen Y (2016). Efficient refolding of the bifunctional therapeutic fusion protein VAS-TRAIL by a triple agent solution. Protein Expr Purif..

[16] Dahiya V, Chaudhuri TK (2014). Chaperones GroEL/GroES accelerate the refolding of a multidomain protein through modulating on-pathway intermediates. J Biol Chem..

[17] de Ruyck J, Brysbaert G, Blossey R, Lensink MF (2016). Molecular docking as a popular tool in drug design, an in silico travel. Adv Appl Bioinform Chem..

[18] Sarroukh R, Goormaghtigh E, Ruysschaert JM, Raussens V (2013). ATR-FTIR: a “rejuvenated” tool to investigate amyloid proteins. Biochim Biophys Acta..

[19] Wiley SR (1995). Identification and characterization of a new member of the TNF family that induces apoptosis. Immunity..

[20] van der Sloot AM, Mullally MM, Fernandez-Ballester G, Serrano L, Quax WJ (2004). Stabilization of TRAIL, an all-beta-sheet multimeric protein, using computational redesign. Protein Eng Des Sel..

[21] Singh A, Upadhyay V, Upadhyay AK, Singh SM, Panda AK (2015). Protein recovery from inclusion bodies of *Escherichia coli* using mild solubilization process. Microb Cell Fact..

[22] Xia XX, Shen YL, Wei DZ (2004). Purification and Characterization of Recombinant sTRAIL Expressed in *Escherichia coli*. Acta Bioch Bioph Sin..

[23] Upadhyay AK, Singh A, Mukherjee KJ, Panda AK (2014). Refolding and purification of recombinant L-asparaginase from inclusion bodies of *E. coli* into active tetrameric protein. Front Microbiol..

[24] Von KS, Montinaro A, Walczak H (2017). Exploring the TRAILs less travelled: TRAIL in cancer biology and therapy. Nat Rev Cancer..

[25] Li P, Gu Q, Wu X (2016). Fed-batch production of tumor necrosis factor-related apoptosis-inducing ligand (TRAIL) in soluble form in *Escherichia coli* and its purification and characterization. Protein Expr Purif..

[26] Jariyachawalid K, Laowanapiban P, Meevootisom V, Wiyakrutta S (2012). Effective enhancement of Pseudomonas stutzeri D-phenylglycine aminotransferase functional expression in Pichia pastoris by co-expressing *Escherichia coli* GroEL-GroES. Microb Cell Fact..

[27] Horwich AL (2014). Molecular chaperones in cellular protein folding: the birth of a field. Cell..

[28] Chen DH (2013). Visualizing GroEL/ES in the act of encapsulating a folding protein. Cell..

[29] Carrió MM, Villaverde A (2005). Localization of chaperones DnaK and GroEL in bacterial inclusion bodies. J Bacteriol..

[30] Carrió MM, Villaverde A (2003). Role of molecular chaperones in inclusion body formation. FEBS Lett..

[31] Zblewska K, Krajewska J, Zolkiewski M, Kedzierska-Mieszkowska S (2014). Role of the disaggregase ClpB in processing of proteins aggregated as inclusion bodies. Arch Biochem Biophys..

[32] Wanner KADBL (2000). One-step inactivation of chromosomal genes in *Escherichia coli* K-12 using PCR products. PNAS..

[33] Wang L (2001). Higher density fermentation in preparation of recombinant soluble human TRAIL. Acad J Sec Mil Med Coll..

[34] Shen YL (2003). Refolding and purification of Apo2L/TRAIL produced as inclusion bodies in high-cell-density cultures of recombinant *Escherichia coli*. Biotechnol Lett..

[35] Braig K, Adams PD, Brünger AT (1995). Conformational variability in the refined structure of the chaperonin GroEL at 2.8 A resolution. Nat Struct Biol..

[36] Hymowitz SG (2000). A unique zinc-binding site revealed by a high-resolution X-ray structure of homotrimeric Apo2L/TRAIL. Biochemistry..

[37] Zhu H, Pan RJ, Wang TW, Shen YL, Wei DZ (2006). Functional solubilization of aggregation-prone TRAIL protein facilitated by coexpressing with protein isoaspartate methyltranferase. Appl Microbiol Biot..

[38] Chura-Chambi RM (2013). An analysis of the factors that affect the dissociation of inclusion bodies and the refolding of endostatin under high pressure. Process Biochem..

[39] Fan J (2015). Strategy for linker selection to enhance refolding and bioactivity of VAS-TRAIL fusion protein based on inclusion body conformation and activity. J Biotechnol..

