# Supplementary material for: GroEL/ES mediated the in vivo recovery of TRAIL inclusion bodies in Escherichia coli
Source: Sci Rep. 2018 Oct 25;8:15766. doi: 10.1038/s41598-018-34090-7 (PMC6202318; doi:10.1038/s41598-018-34090-7)

**GroEL/ES mediated the *in vivo* re****covery of TRAIL inclusion bodies in *Escherichia coli***

Zhanqing Wang^1^, Min Zhang^1^, Xin Lv^1^, Jiying Fan^1^, Jian Zhang^1^, Jing Sun^1,2^ and Yaling Shen^1*^

^1^State Key Laboratory of Bioreactor Engineering, Shanghai Collaborative Innovation Center for Biomanufacturing Technology, East China University of Science and Technology, Shanghai, People’s Republic of China;

^2^Shanghai Gebaide Biotechnical Co., Ltd., Shanghai, People’s Republic of China

*Corresponding authors:

Mailing address for Prof. Yaling Shen: State Key Laboratory of Bioreactor Engineering, East China University of Science and Technology, Shanghai 200237, People’s Republic of China, E-mail: [ylshen@ecust.edu.cn](mailto:ylshen@ecust.edu.cn), Tel.: +86-2164253156; Fax: +86-2164250068.

**Supplementary Figure S1** Growth curves of different strains. The curves for wt, GroEL/ES^+^ and GroEL/ES^-^ are represented by squares, circles and triangles, respectively. Data are presented as the mean ± SD (*n* = 3; * *p* < 0.05 versus the wt strain).





**Supplementary Figure S2** Growth of the wt, GroEL/ES^+^ and GroEL/ES^-^ strains. CFU indicates colony forming unit. Data are presented as the mean ± SD (n = 3; **p* < 0.05 and ***p* < 0.01 versus the wt strain).


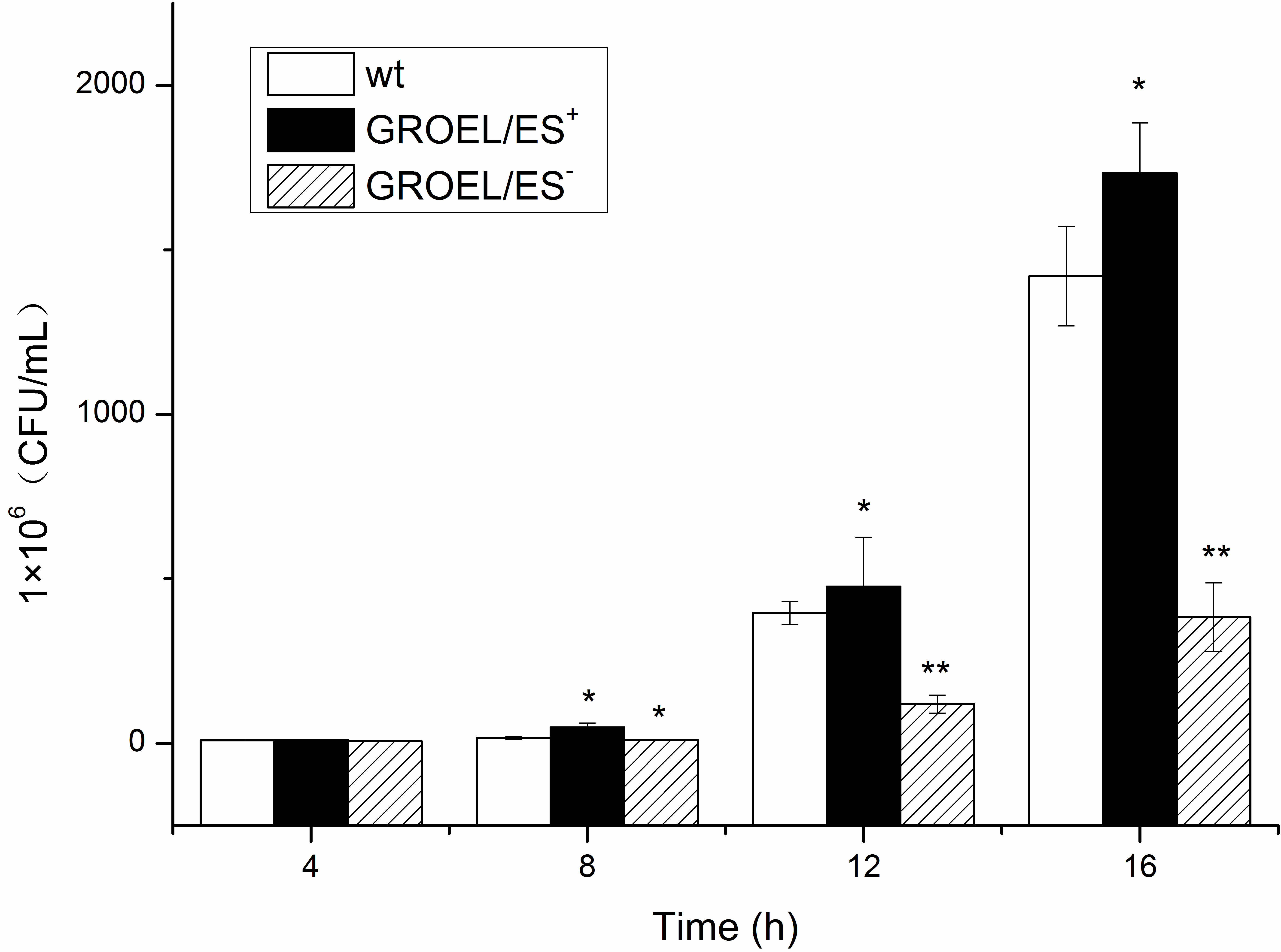


**Supplementary Figure S3** Images of *E. coli* plates. Different strains are indicated on the right. The circles on the GroEL/ES^+^ and wt plates are samples diluted 10^-1^-10^-4^, 10^-2^-10^-5^,10^-3^-10^-6^ and 10^-4^-10^-7^ from the top to the bottom. The circles on the GroEL/ES^-^ plates are samples diluted 10^-1^-10^-4^, 10^-2^-10^-5^,10^-2^-10^-5^ and 10^-3^-10^-6^ from the top to the bottom. Each sample was tested three times in triplicate.


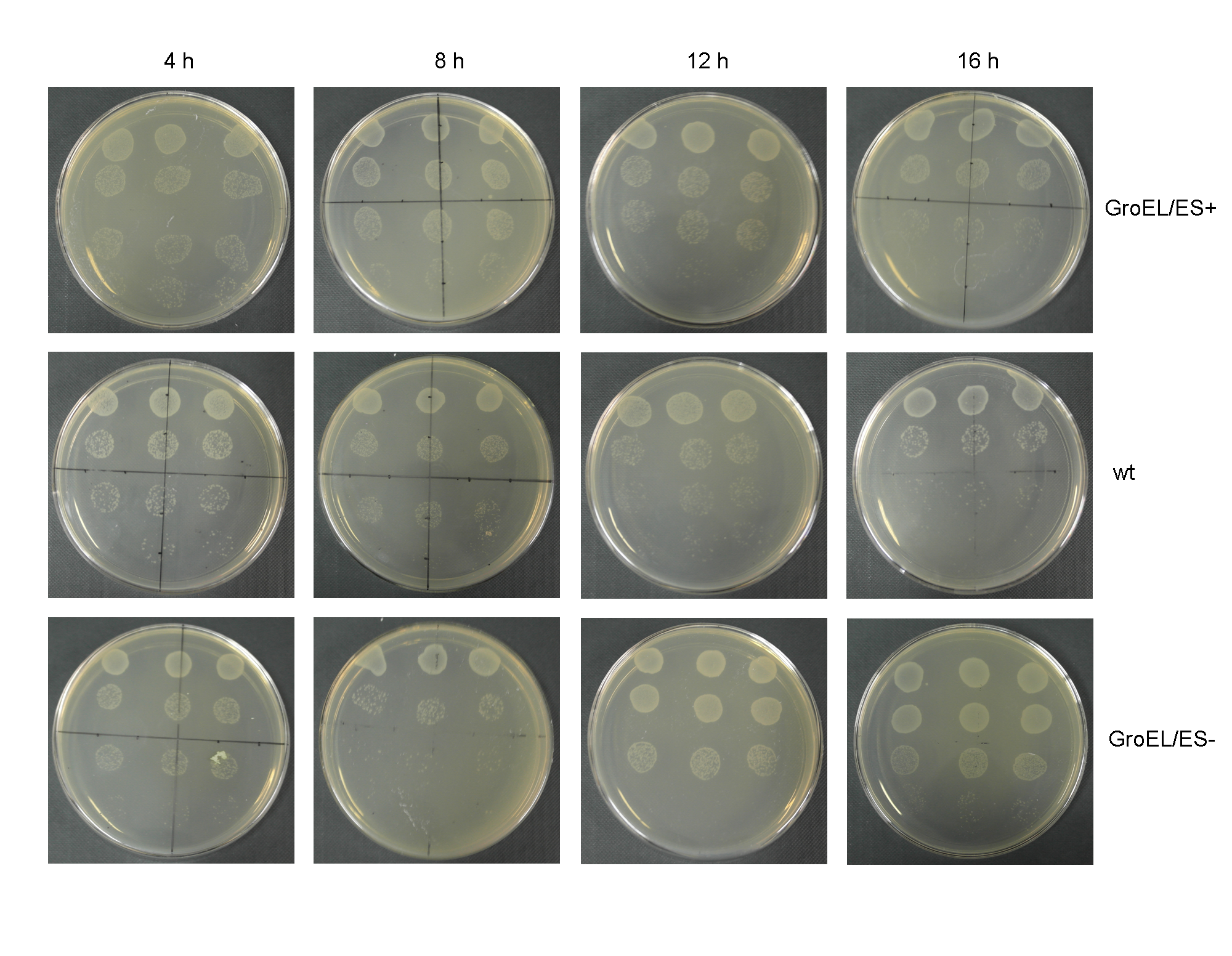


**Supplementary Figure S4** Full-length SDS-PAGE gel of rhTRAIL expression in different strains.


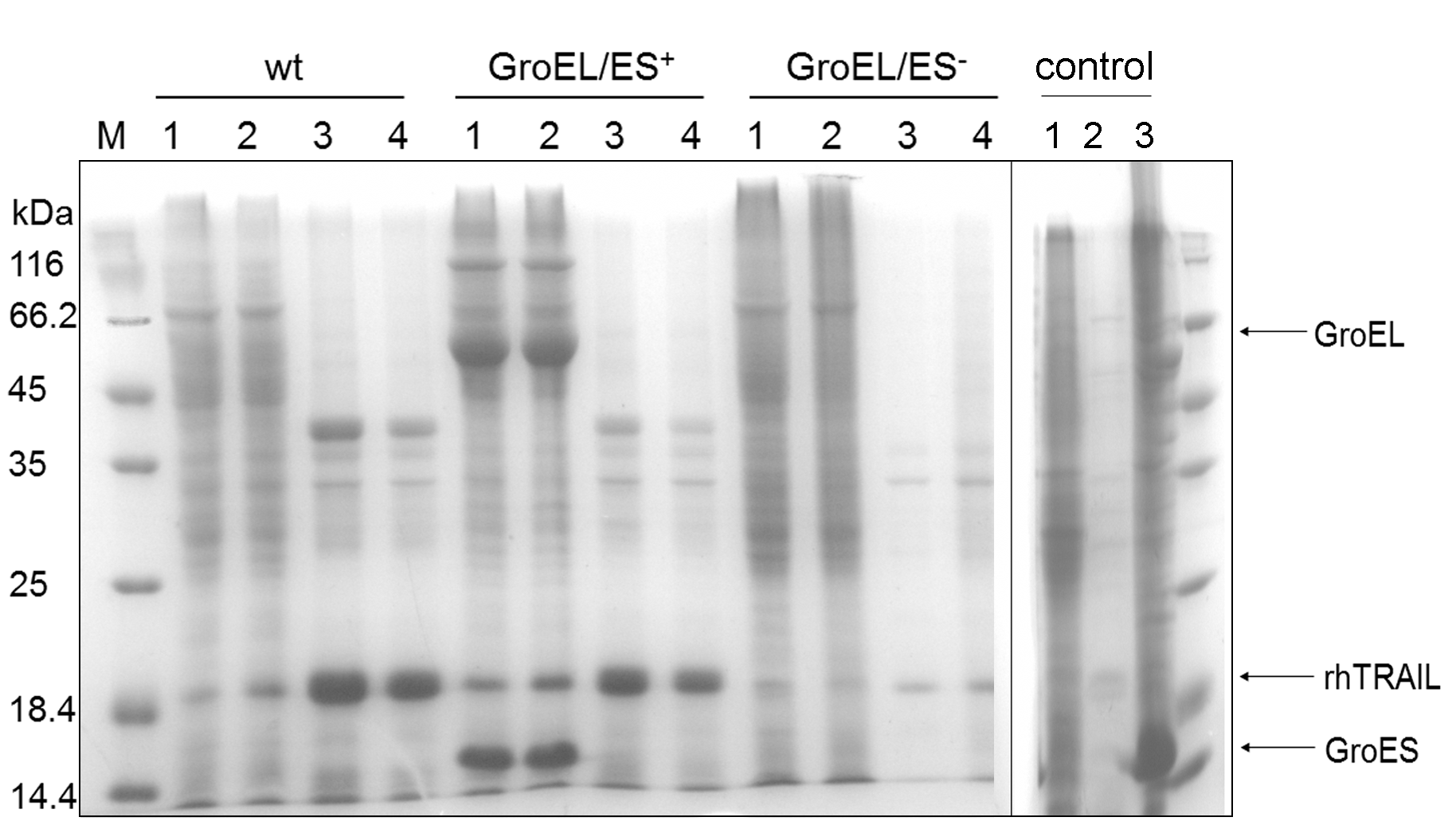


**Supplementary Figure S5** Full-size images of Western blots to detect GroEL/ES (A) and rhTRAIL (B) in different strains, corresponding to Figure 1.

*
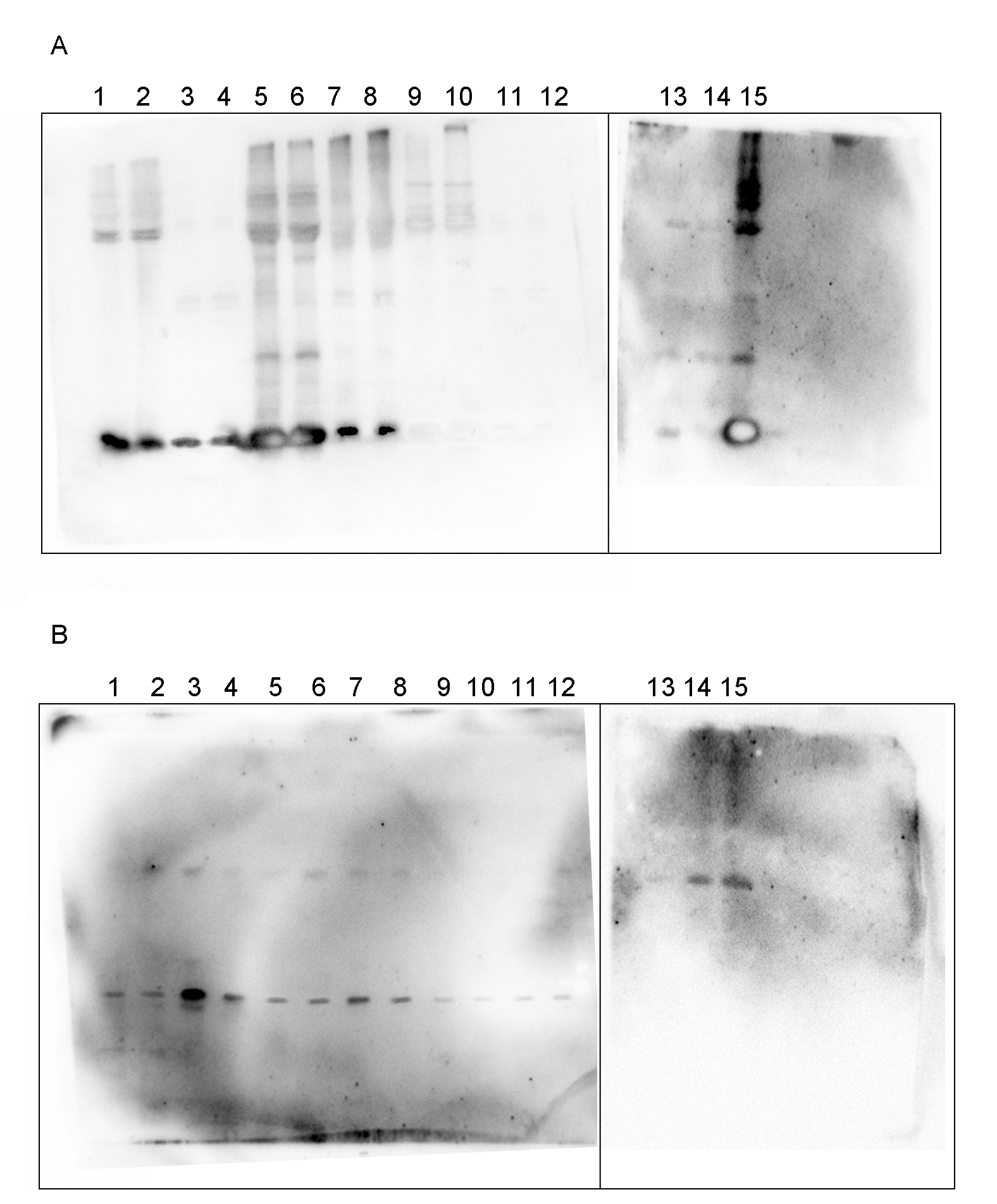
*

**Supplementary Figure S6** Full-size images of Western blots with antibodies against GroEL (A) and TRAIL (B), corresponding to Figure 3.

*
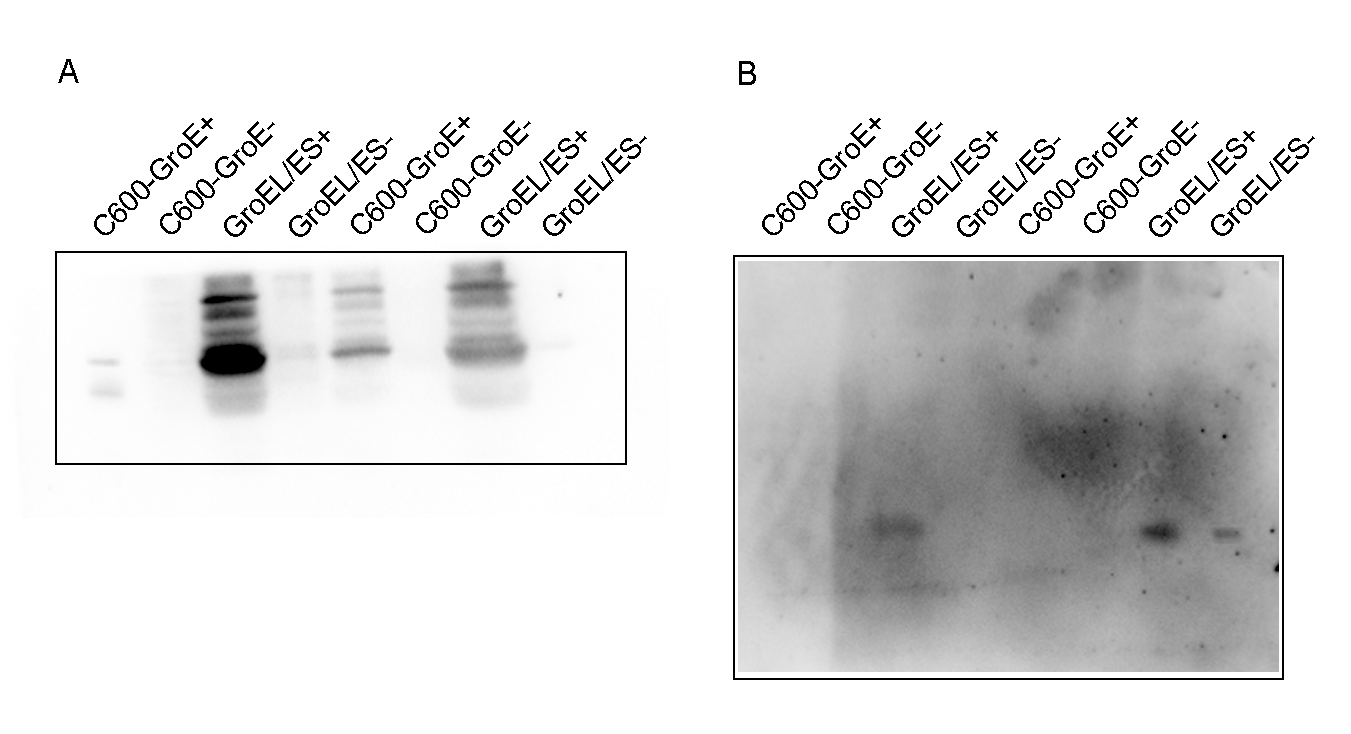
*

**Supplementary Figure S7** Raw FT-IR data for IBs produced by the wt, GroEL/ES^+^ and GroEL/ES^-^ strains. Spectra of samples before and after *in vivo* refolding are shown. CT, CGT and DGT represent IBs produced by the wt, GroEL/ES^+^ and GroEL/ES^-^ strains, respectively. CT', CGT' and DGT' are the corresponding IBs produced after *in vivo* refolding. The amide I (1700-1600 cm^-1^) and amide II regions (1600-1500 cm^-1^) of FTIR spectra were marked with dotted lines.


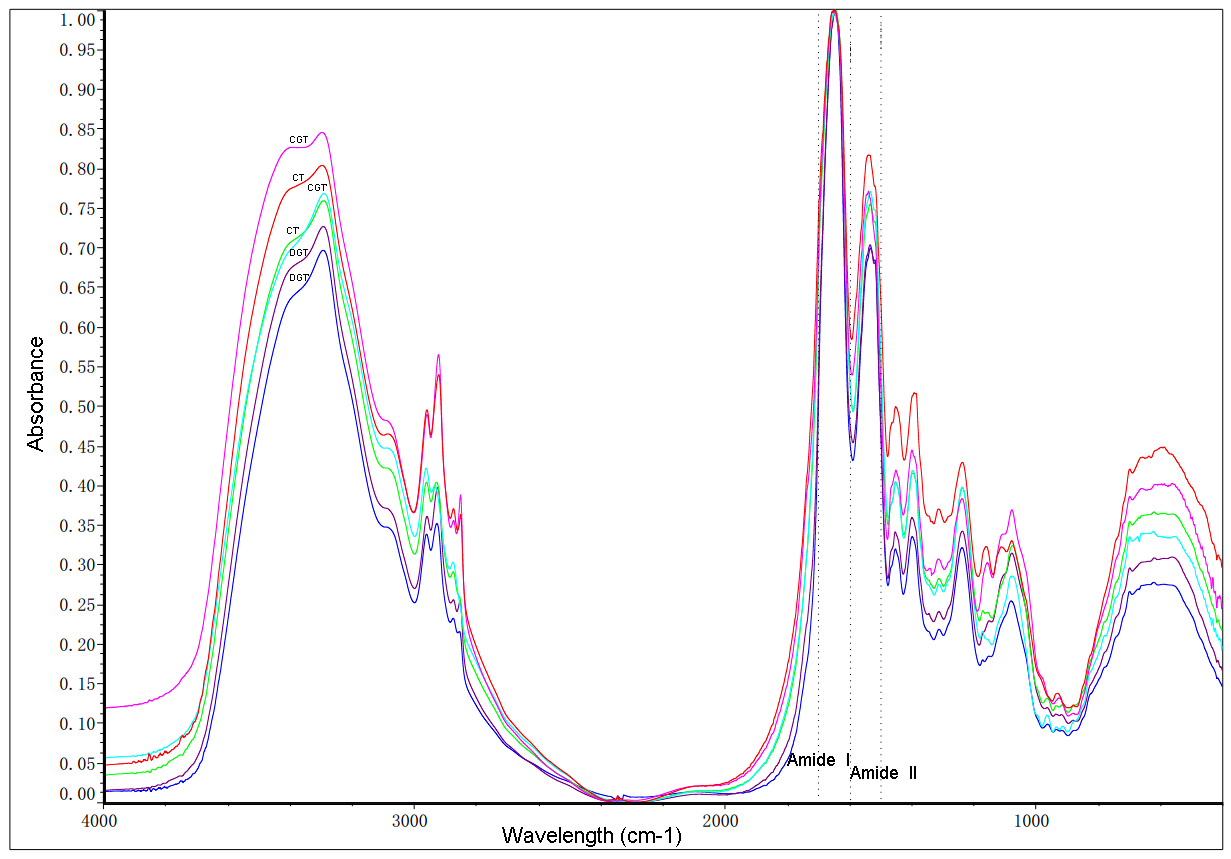

Supplement: Supplementary file 1 — Supplementary Information [file 41598_2018_34090_MOESM1_ESM.doc]
